# Incidence of genitourinary complications following radiation therapy for localised prostate cancer

**DOI:** 10.1007/s00345-022-04124-x

**Published:** 2022-08-11

**Authors:** Rowan V. David, Arman A. Kahokehr, Jason Lee, David I. Watson, John Leung, Michael E. O’Callaghan

**Affiliations:** 1 College of Medicine and Public Health, Flinders University, Bedford Park, SA Australia; 2 Department of Urology, Flinders Medical Centre, SA Health, Bedford Park, Australia; 3 GenesisCare, Adelaide, Australia; 4 South Australian Prostate Cancer Clinical Outcomes Collaborative, Adelaide, Australia; 5 Discipline of Medicine, Freemasons Foundation Centre for Men’s Health, University of Adelaide, Adelaide, Australia

**Keywords:** Prostate cancer, Radiotherapy, Radiation therapy, External beam radiotherapy, Genitourinary complications, Urethral stricture, Radiation cystitis

## Abstract

**Purpose:**

Studies of genitourinary toxicity following radiotherapy for prostate cancer are mainly from high volume single institutions and the incidence and burden of treatment remain uncertain. Hence we determine the cumulative incidence of treatment-related genitourinary toxicity in patients with localised prostate cancer treated with primary external beam radiotherapy (EBRT) at a state population level.

**Methods:**

We analysed data from a prospective population-based cohort, including hospital admission and cancer registry data, for men with localised prostate cancer who underwent primary EBRT without nodal irradiation between 1998 and 2019 in South Australia. The 10-year cumulative incidence of genitourinary toxicity requiring hospitalisation or procedures was determined. Clinical predictors of toxicity and the volume of admissions, non-operative, minor operative and major operative procedures were determined.

**Results:**

All the included patients (*n* = 3350) had EBRT, with a median (IQR) of 74 Gy (70–78) in 37 fractions (35–39). The 10-year cumulative incidence of was 28.4% (95% CI 26.3–30.6) with a total of 2545 hospital admissions, including 1040 (41%) emergency and 1893 (74%) readmissions. The 10-year cumulative incidence of patients in this cohort requiring a urological operative procedure was 18% (95% CI 16.1–19.9), with a total of 106 (4.2%) non-operative, 1044 (41%) minor operative and 57 (2.2%) major operative urological procedures.

**Conclusions:**

Genitourinary toxicity after radiotherapy for prostate cancer is common. Although there continue to be advancements in radiotherapy techniques, patients and physicians should be aware of the risk of late toxicity when considering EBRT.

**Supplementary Information:**

The online version contains supplementary material available at 10.1007/s00345-022-04124-x.

## Introduction

Prostate cancer is the second most common form of cancer affecting men worldwide [1]. The majority (94%) of patients with prostate cancer have curable localised disease, for which the treatment options include active surveillance, surgery or radiotherapy [2]. Radiotherapy is a common treatment for localised prostate cancer [3, 4]. However, the incidence of late genitourinary toxicity (GUT) and its associated burden of treatment across a variety of practice settings remains poorly understood. Radiotherapy injuries often present late due to progressive fibrosis and the difficulties in accurately recording these long-term adverse effects are reported in the literature frequently [5–7]. The majority of studies on the incidence of genitourinary toxicity after radiotherapy and its associated burden of treatment are studies from specialised high-volume single centres [7–9]. There are few multi-institutional studies [10–12] and the randomised trials often involve a disproportionately younger and healthier patient demographic when compared to a typical population [13, 14]. An improved understanding of the incidence of late treatment-related genitourinary toxicity following prostate radiotherapy would enhance patient-centred decision making [6].

The primary aim of this study was to determine the cumulative incidence of treatment-related genitourinary toxicity following external beam prostatic radiotherapy in patients with localised prostate cancer at a population level. The secondary aims were to determine clinical factors predictive of genitourinary toxicity and the volume of admissions and procedures required.

## Materials and methods

### Participants

**Fig. 1:**
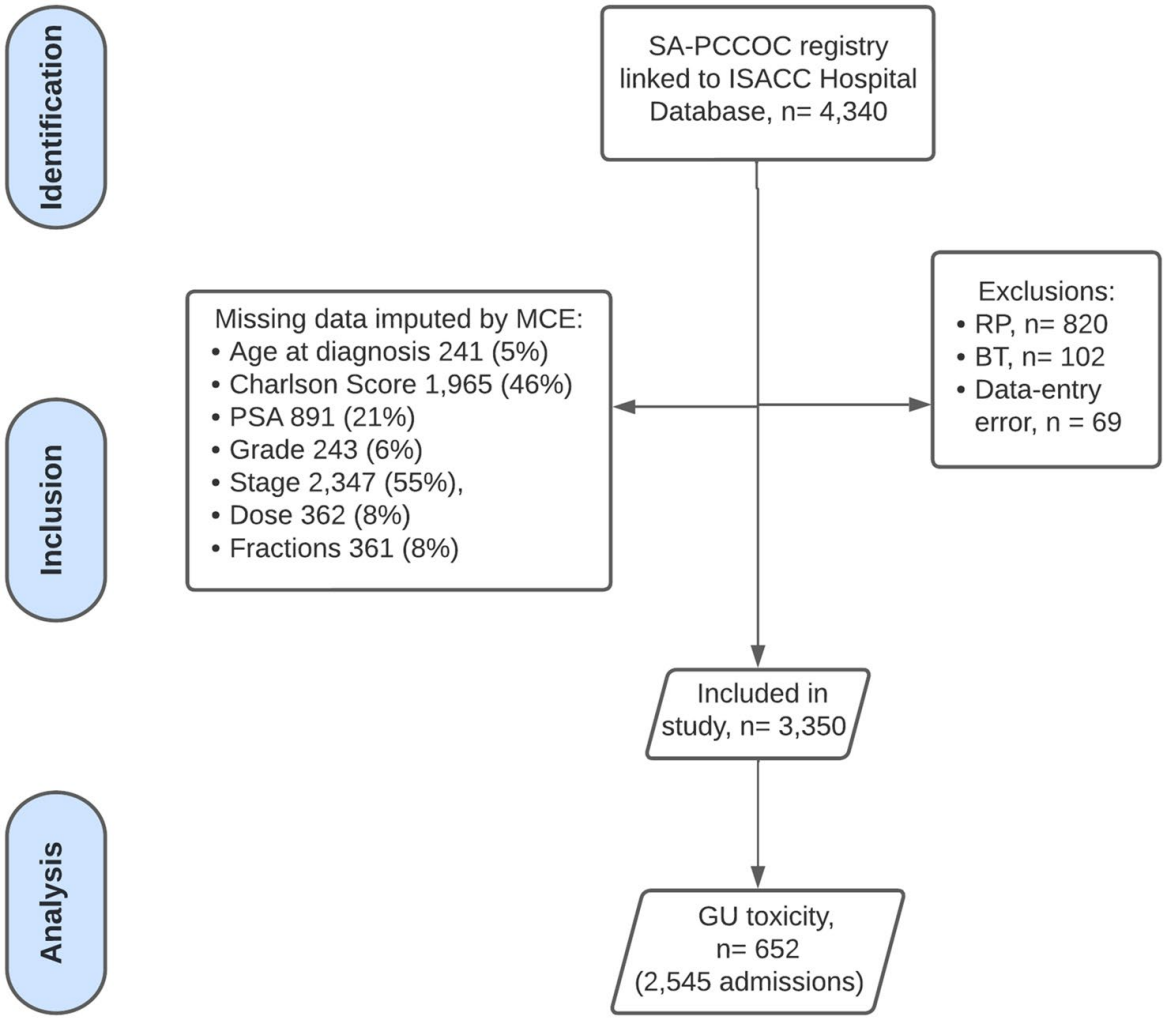
HYPERLINK "sps:id::fig1||locator::gr1||MediaObject::0" Patient selection flow chart. SA-PCCOC, South Australian Prostate Cancer Clinical Outcomes Collaborative; ISAAC, Integrated South Australian Activity Collection; RP, radical prostatectomy; BT, brachytherapy; GUT, genitourinary toxicity

A population-based prospective cohort study of all patients with localised (T1–T3, according to the American Joint Committee on Cancer) biopsy-proven prostate cancer who underwent primary external beam radiotherapy (EBRT) was performed between January 1, 1998, and January 31, 2019, in South Australia. We excluded patients with metastatic prostate cancer and those without a histological tissue diagnosis of prostate cancer. We excluded patients who underwent adjuvant radiotherapy following either radical prostatectomy, or prior radiotherapy treatment (Fig. 1).

The South Australian Prostate Cancer Clinical Outcome Collaborative (SA-PCOCC) registry prospectively recruits > 90% of patients who are diagnosed with prostate cancer in South Australia. We linked patient records from the SA-PCCOC registry with the Integrated South Australian Activity Collection (ISAAC) Hospital Administrative Database to identify patients who presented to any major hospital in South Australia with treatment-related genitourinary toxicity, as defined by a pre-selected list of International Classification Disease 10th Edition (ICD-10-AM)/Australian Classification of Health Interventions (ACHI). Data linkage was performed by matching patient identifiers within Envido (Adelaide, South Australia). The list of admission and procedures codes were selected based on the literature [6], and recommendations from a multidisciplinary panel, including a urologist, radiation oncologist, general surgeon and a clinical epidemiologist (Appendix 1). Baseline characteristics including age, Charlson Comorbidity Index, anticoagulant medication use, and oncological characteristics, including T-stage, Gleason score and baseline Prostate-specific antigen (PSA) level were extracted. Treatment-related factors including dose (Gray), fractionation and date of treatment completion were also extracted.

### Primary outcomes

The treatment-related complication categories used were hospital admission and urological procedures associated with genitourinary toxicity. Genitourinary toxicity-related hospital admission or procedures required for each patient were identified using the ISAAC Database (using the relevant hospital admission or procedure code based on the ICD-10 or ACHI codes). The time to the first genitourinary toxicity-related hospital admission, death or censor were analysed to determine the cumulative incidence of genitourinary toxicity. Patients were censored at the last date of the last admission in the ISAAC electronic hospital database.

### Secondary outcomes

Demographic factors assessed included age (continuum), Charlson comorbidity score, diabetes (yes/no), hypertension (yes/no), use of anticoagulant (yes/no), smoking history (yes/no), bladder outlet obstruction (yes/no), Transurethral resection of the prostate (TURP) before radiotherapy (yes/no), T stage (T1 vs T2 vs T3), initial prostate-specific antigen level (continuum) and dose (continuum and > 80 Gy vs ≤ 80 Gy). Furthermore, the admission data was separated into patients who received EBRT < 2009 and ≥ 2009, to account for the use of Three-dimensional conformal radiation therapy (3DCRT) and Intensity Modulated Radiotherapy/Volumetric modulated arc therapy (IMRT/VMAT), respectively.

The overall burden of treatment, as defined by the volume of admissions as well as non-operative, minor operative and major operative procedures was determined. Non-operative procedures were defined as ACHI codes involving urethral catheterization or bladder irrigation. Minor operative procedures were defined as ACHI codes involving urethral dilation, cystoscopy, suprapubic catheter insertion, retrograde pyelogram, antegrade or retrograde ureteric stenting. Major operative procedures were defined as ACHI codes involving transurethral resection, ureteroscopy or open surgical procedure.

The outcomes were reported according to the Strengthening the Reporting of Observational Studies in Epidemiology (STROBE) statement [15].

### Statistical analysis

The cumulative incidence of hospitalisation for treatment-related genitourinary complications was determined. Patients were considered to be at risk of complications from the end date of their radiotherapy until either the date of their first admission related to genitourinary toxicity, last date of follow-up or date of death, according to the SA-PCCOC registry. The patient-related baseline characteristics and the volume of hospital admissions and procedures were summarised and compared. Categorical variables were compared using the Fischer Exact Test or Pearson’s Chi-square test. Continuous parametric and non-parametric variables were compared using one-way ANOVA or the Kruskal–Wallis Rank Sum test, respectively. *p* values were calculated for each variable compared and *p* < 0.05 was considered significant. Relationships between genitourinary toxicity-related hospital admission and patient, tumour or treatment characteristics were analysed using Cox proportional hazard regression at univariate and multivariate levels. The regression analyses’ results are presented as a hazard with a 95% confidence interval. Missing clinical data was replaced using multiple imputations by chained equations before regression analysis (Fig. 1). All statistical analysis was performed using R language, Version 3.2.1 (R Foundation for Statistical Computing, Vienna, Austria) [16].

## Results

There were 3350 patients with prostate cancer treated with primary external beam radiotherapy in this cohort. We excluded 820 patients who were initially treated surgically, with either robotic-assisted laparoscopic prostatectomy (*n* = 579) or open radical prostatectomy (*n* = 241). We also excluded 388 patients who were treated with brachytherapy before external beam radiotherapy and four patients with T4 disease (Fig. 1). All the included patients underwent primary EBRT, with a median (IQR) of 74 Gy (70–78) in 37 fractions (35–39). The median (IQR) age at diagnosis of the included patients was 71 (66–76). Most patients had Stage II (*n* = 914, 58%) and high-risk disease (*n* = 1517 [51%]), according to the National Comprehensive Cancer Network (NCCN) 2017 scoring system. Table 1 summarises the patient demographic, oncological and treatment dosimetric characteristics.

**Table 1:** Baseline characteristics of all included prostate cancer patients treated with primary radiotherapy

Characteristic	Overall, *N* = 3350^1^	GU toxicity admission		*p* value^2^
		No, *N* = 2698^1^	Yes, *N* = 652^1^	
Age at diagnosis				> 0.9^†^
Median (IQR)	71 (66, 76)	71 (66, 76)	72 (66, 75)	
Range	43, 91	43, 91	47, 86	
(Missing)	152	127	25	
Charlson score				0.14^‡^
1. 0	1161/1856 (63%)	930/1457 (64%)	231/399 (58%)	
2. 1–2	546/1856 (29%)	415/1457 (28%)	131/399 (33%)	
3. 3–4	122/1856 (6.6%)	90/1457 (6.2%)	32/399 (8.0%)	
4. > 4	27/1856 (1.5%)	22/1457 (1.5%)	5/399 (1.3%)	
(Missing)	1494	1241	253	
Diabetes				**< 0.001**^**‡**^
No	2697/3349 (81%)	2228/2697 (83%)	469/652 (72%)	
Yes	652/3349 (19%)	469/2697 (17%)	183/652 (28%)	
Anticoagulation				**< 0.001**^**‡**^
No	3044/3349 (91%)	2509/2697 (93%)	535/652 (82%)	
Yes	305/3349 (9.1%)	188/2697 (7.0%)	117/652 (18%)	
Smoking				**< 0.001**^**‡**^
No	1768/3349 (53%)	1576/2697 (58%)	192/652 (29%)	
Yes	1581/3349 (47%)	1121/2697 (42%)	460/652 (71%)	
TURP before RT				**< 0.001**^**‡**^
No	2925/3349 (87%)	2,463/2697 (91%)	462/652 (71%)	
Yes	424/3349 (13%)	234/2697 (8.7%)	190/652 (29%)	
(Missing)	738	624	114	
Tumour stage				0.3^‡^
1	458/1577 (29%)	358/1196 (30%)	100/381 (26%)	
2	914/1577 (58%)	682/1196 (57%)	232/381 (61%)	
3	205/1577 (13%)	156/1196 (13%)	49/381 (13%)	
Gleason grade				**< 0.001**^**‡**^
1. < 7	975/3113 (31%)	744/2510 (30%)	231/603 (38%)	
2. 3 + 4	775/3113 (25%)	643/2510 (26%)	132/603 (22%)	
3. 4 + 3	568/3113 (18%)	471/2510 (19%)	97/603 (16%)	
4. > 7	795/3113 (26%)	652/2510 (26%)	143/603 (24%)	
(Missing)	237	188	49	
Baseline BOO				**< 0.001**^**‡**^
No	2909/3349 (87%)	2551/2697 (95%)	358/652 (55%)	
Yes	440/3349 (13%)	146/2697 (5.4%)	294/652 (45%)	
Radiotherapy dose, Gy*				**< 0.001**^**‡**^
1. < 74	1473/3350 (44%)	1119/2698 (41%)	354/652 (54%)	
2. ≥ 74	1877/3350 (56%)	1579/2698 (59%)	298/652 (46%)	
Fractions*				**< 0.001**^**†**^
Median (IQR)	37 (35, 39)	37 (35, 39)	37 (35, 37)	
Range	20, 42	20, 42	20, 42	
Radiotherapy date				**< 0.001**^**‡**^
< 2009	1499/3345 (45%)	1038/2693 (39%)	461/652 (71%)	
≥ 2009	1846/3345 (55%)	1655/2693 (61%)	191/652 (29%)	
(Missing)	5	5	0	
Follow-up, years				**< 0.001**^**†**^
Median (IQR)	5.33 (2.10, 9.01)	5.73 (2.29, 9.48)	3.66 (1.59, 7.03)	
Range	0.01, 20.68	0.01, 20.68	0.01, 18.39	
(Missing)	107	99	8	
Status				**< 0.001**^**‡**^
1. Alive	2205/3349 (66%)	1945/2697 (72%)	260/652 (40%)	
2. Died from Prostate cancer	382/3349 (11%)	230/2697 (8.5%)	152/652 (23%)	
3. Died from other cause	762/3349 (23%)	522/2697 (19%)	240/652 (37%)	
(Missing)	1	1	0	

Any statistically significant *p*-values were bolded

GU, Genitourinary; TURP, Transurethral Resection of Prostate; PSA, Prostate-specific antigen; NCCN, National Comprehensive Cancer Network; BOO, bladder outlet obstruction; Gy, Gray

^1^*n*/*N* (%)

^2^Wilcoxon rank sum test^†^; Pearson’s Chi-squared test^‡^; Fisher’s exact test^±^

^*^Missing data imputed by multiple chained equations: Radiotherapy Dose, Gy (*n* = 311 [no event: 236; event: 75]), Fractions, (*n* = 311 [no event: 234; event: 77])

**Fig. 2:**
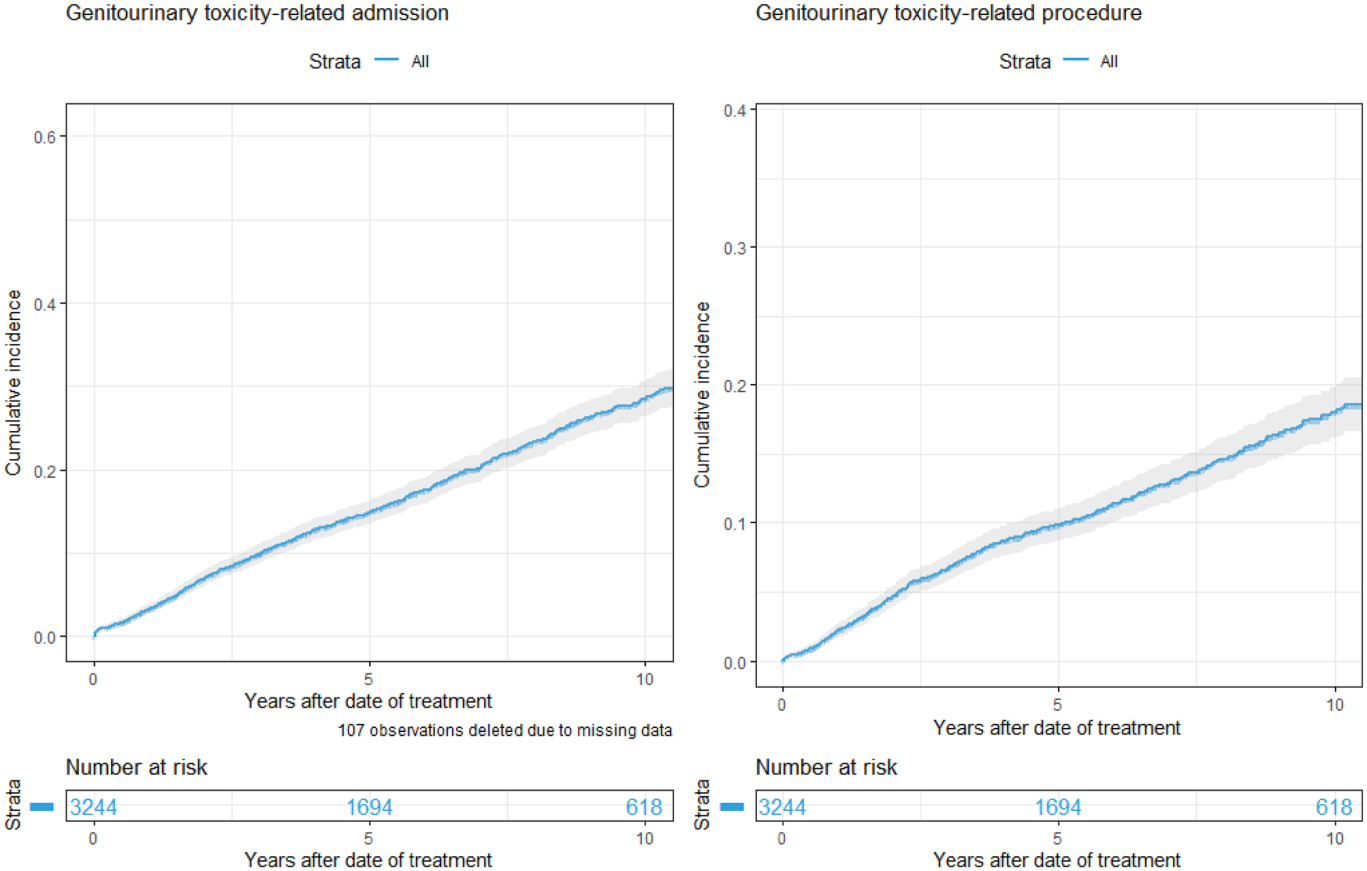
10-year cumulative incidence of admission or urological procedure (non-operative or operative) for genitourinary toxicity following prostate radiotherapy

**Fig. 3:**
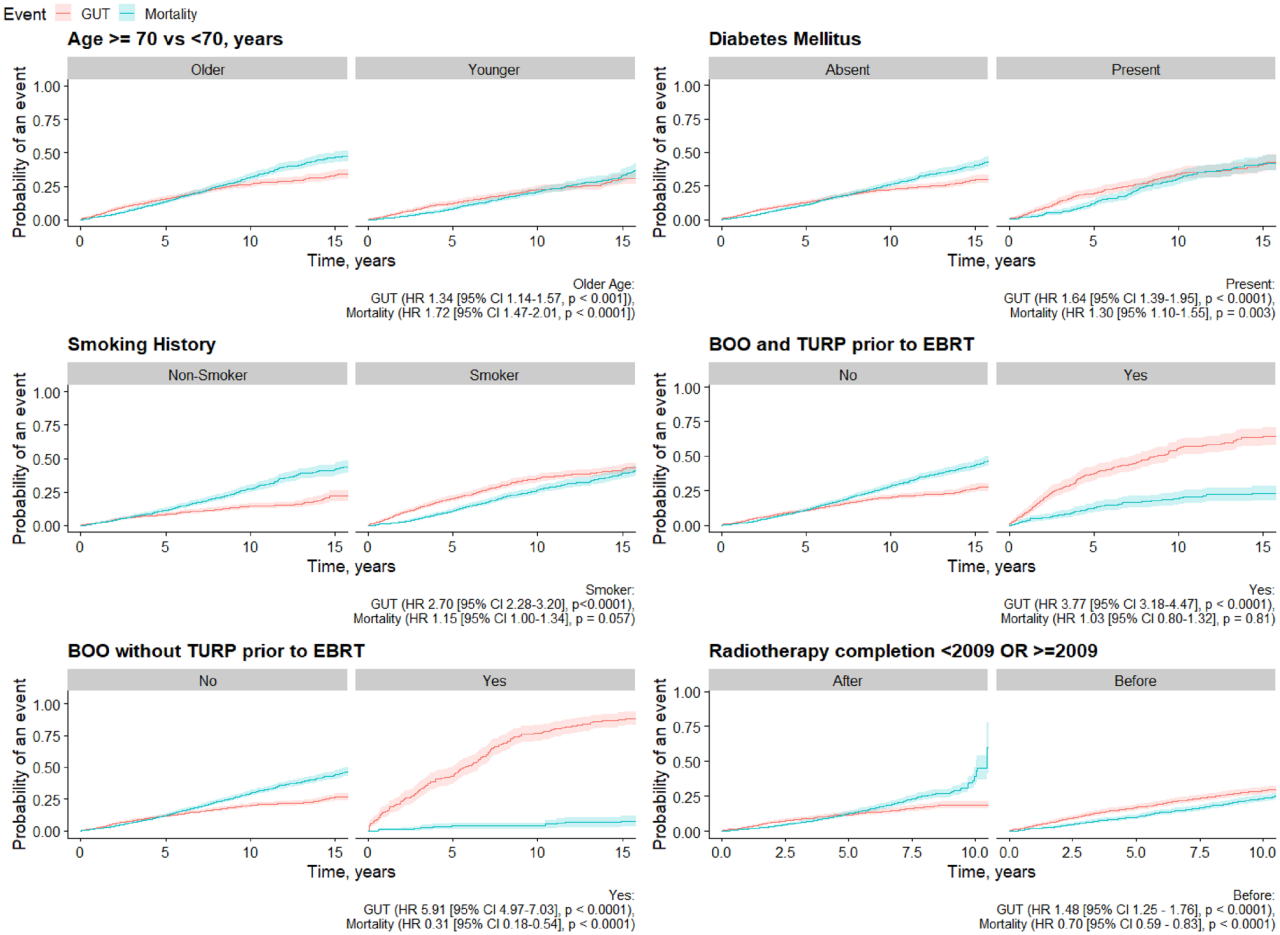
10-year cumulative incidence of admission for genitourinary toxicity following prostate EBRT by clinical factors

The 5- and 10-year cumulative incidence of admission to hospital for treatment-related genitourinary toxicity were 14.8% (95% CI 13.4–16.2) and 28.4% (95% CI 26.3–30.6), respectively (Fig. 2). The 5- and 10-year cumulative incidence of patients in this cohort requiring a urological operative procedure for a treatment-related GUT were 9.9% (95% CI 8.7–11) and 18% (95% CI 16.1–19.9), respectively (Fig. 2). The 5-year cumulative incidence of treatment-related genitourinary toxicity hospital admission were 18% (95% CI 15–20%) and 12% (95 CI 11–14), amongst patients treated before and after 2010, respectively (*p* < 0.001; Fig. 3).

There were 652 (19.5%) prostate cancer patients who required hospital admission for genitourinary toxicity after primary EBRT, with a total of 2545 hospital admissions, of which 1040 (41%) occurred in the emergency setting. Four-hundred and nine (63%) of these patients had multiple admissions, with a total of 1893 (74%) readmission related to genitourinary toxicity. Haematuria was the most common genitourinary toxicity (*n* = 386, 59%), and of these patients, 108 (28%) required blood product transfusion, 8 (2%) required HBOT and 4 (1%) required surgical urinary diversion. Table 2 summarises the treatment-related outcomes amongst patients with genitourinary toxicity following primary EBRT. Four-hundred and nine (12%) patients developed genitourinary toxicity which required management with a urological procedure, with a total of 106 (4.2%) non-operative, 1044 (41%) minor and 57 (2.2%) major operative urological procedures (Table 3). The most common procedure was diagnostic cystoscopy (701/1101 [64%] of all procedures).

**Table 2:** Volume of admissions and procedures for genitourinary toxicity amongst included prostate cancer patients treated with primary prostate radiotherapy

Outcome	Overall, *N* = 2545^1^	Date of radiotherapy		*p* value^2^
		1. < 2009, *N* = 1781^1^	2. ≥ 2009, *N* = 764^1^	
GU toxicity admission	2545/2545 (100%)	1781/1781 (100%)	764/764 (100%)	
Admission category				0.39
1. Elective	1505/2545 (59%)	1063/1781 (60%)	442/764 (58%)	
2. Emergency	1040/2545 (41%)	718/1781 (40%)	322/764 (42%)	
GU toxicity readmission	1893/2545 (74%)	1364/1781 (77%)	529/764 (69%)	**< 0.001**
Healthcare sector				**< 0.001**
Private	231/2545 (9.1%)	135/1781 (7.6%)	96/764 (13%)	
Public	2314/2545 (91%)	1646/1781 (92%)	668/764 (87%)	
Length of stay, mean (SD)	4.76 (9.96)	4.59 (9.64)	5.13 (10.65)	0.24
Haematuria	1509/2545 (59%)	1049/1781 (59%)	460/764 (60%)	0.54
Obstruction	1045/2545 (41%)	753/1781 (42%)	292/764 (38%)	0.056
Incontinence	355/2545 (14%)	251/1781 (14%)	104/764 (14%)	0.75
No procedure	1402/2545 (55%)	934/1781 (52%)	468/764 (61%)	**< 0.001**
Non-operative procedure	106/2545 (4.2%)	83/1781 (4.7%)	23/764 (3.0%)	0.056
Minor operative procedure	1044/2545 (41%)	769/1781 (43%)	275/764 (36%)	**< 0.001**
Major operative procedure	57/2545 (2.2%)	41/1781 (2.3%)	16/764 (2.1%)	0.75
Diagnostic cystoscopy	701/2545 (28%)	499/1781 (28%)	202/764 (26%)	0.41
Urethral dilation	198/2545 (7.8%)	152/1781 (8.5%)	46/764 (6.0%)	**0.030**
Cystoscopic washout	157/2545 (6.2%)	111/1781 (6.2%)	46/764 (6.0%)	0.84
Ureteric stent procedure	101/2545 (4.0%)	84/1781 (4.7%)	17/764 (2.2%)	**0.003**
Suprapubic catheter	70/2545 (2.8%)	62/1781 (3.5%)	8/764 (1.0%)	**< 0.001**
Antegrade percutaneous procedure	17/2545 (0.7%)	15/1781 (0.8%)	2/764 (0.3%)	0.10
TURBT	27/2545 (1.1%)	20/1781 (1.1%)	7/764 (0.9%)	0.64
Bladder repair	11/2545 (0.4%)	4/1781 (0.2%)	7/764 (0.9%)	**0.021**
Urinary diversion	6/2545 (0.2%)	6/1781 (0.3%)	0/764 (0%)	0.19
Ureteric dilation	7/2545 (0.3%)	5/1781 (0.3%)	2/764 (0.3%)	> 0.99
Artificial urinary sphincter	5/2545 (0.2%)	5/1781 (0.3%)	0/764 (0%)	0.33
Ureteric reimplantation	1/2545 (< 0.1%)	1/1781 (< 0.1%)	0/764 (0%)	> 0.99

Any statistically significant *p*-values were bolded

GU, Genitourinary; LOS, Length of stay; TURBT, Transurethral Resection of Bladder Tumour

^1^*n*/*N* (%); Mean(SD)

^2^Pearson’s Chi-squared test; Wilcoxon rank sum test; Fisher’s exact test

**Table 3:** Cumulative Incidence of GU toxicity admission with competing risk regression including overall mortality

Group	Characteristic	Years 5	Years 10	*p* value^1^
GU toxicity	Age ≥ 70 years			**0.017**
	Older	16% (14%, 17%)	26% (24%, 29%)	
	Younger	12% (10%, 14%)	23% (20%, 25%)	
Mortality	Age ≥ 70 years			**< 0.001**
	Older	13% (12%, 15%)	32% (29%, 35%)	
	Younger	8.0% (6.5%, 9.7%)	21% (18%, 24%)	
GU toxicity	Diabetes Mellitus			
	Absent	13% (11%, 14%)	22% (20%, 24%)	**< 0.001**
	Present	20% (16%, 23%)	34% (29%, 38%)	
Mortality	Diabetes Mellitus			0.3
	Absent	11% (9.7%, 12%)	26% (24%, 28%)	
	Present	12% (9.1%, 15%)	31% (26%, 35%)	
GU toxicity	Smoking history			**< 0.001**
	Non-smoker	8.3% (6.9%, 9.8%)	15% (13%, 17%)	
	Smoker	20% (18%, 22%)	35% (32%, 38%)	
Mortality	Smoking history			0.2
	Non-smoker	11% (9.8%, 13%)	27% (25%, 30%)	
	Smoker	11% (9.2%, 13%)	27% (24%, 29%)	
GU toxicity	BOO and TURP prior to EBRT			**< 0.001**
	No	11% (9.5%, 12%)	20% (18%, 22%)	
	Yes	37% (32%, 42%)	56% (50%, 61%)	
Mortality	BOO and TURP prior to EBRT			**< 0.001**
	No	11% (9.7%, 12%)	28% (26%, 30%)	
	Yes	13% (9.5%, 17%)	19% (15%, 24%)	
GU toxicity	BOO without TURP before EBRT			**< 0.001**
	No	12% (10%, 13%)	20% (18%, 22%)	
	Yes	43% (36%, 50%)	77% (70%, 82%)	
Mortality	BOO without TURP before EBRT			**< 0.001**
	No	12% (11%, 13%)	29% (27%, 31%)	
	Yes	3.8% (1.8%, 7.0%)	3.8% (1.8%, 7.0%)	
GU toxicity	EBRT ≥ 2009			**< 0.001**
	After	12% (10%, 14%)	19% (16%, 21%)	
	Before	17% (15%, 19%)	29% (26%, 31%)	
Mortality				**< 0.001**
	After	13% (11%, 14%)	39% (33%, 46%)	
	Before	10.0% (8.4%, 12%)	23% (21%, 26%)	

Any statistically significant *p*-values were bolded

Patients with BOO without TURP prior to EBRT, had the highest 10-cumulative incidence of admission for genitourinary toxicity (77% [70%, 82%] vs 20% [18%, 22%] *p* < 0.001; Table 3, Fig. 3). In addition, patients with BOO without TURP prior to EBRT had the most hospital admissions (178/246 [72%] vs 474/3104 [15%], *p* < 0.001), emergency admissions (136/246 [55%] vs 273/3104 [8.8%], *p* < 0.001) and readmissions (110/246 [45%] vs 282/3104 [9.1], *p* < 0.001), for treatment-related genitourinary toxicity (Table 1). Patients with BOO without TURP before EBRT were at the highest risk of developing genitourinary toxicity after adjustment for age, diabetes, smoking, urinary incontinence and EBRT before 2009 (HR 5.87 [95% CI 4.8–7.17], *p* < 0.001; Table 4).

**Table 4:** Cox proportional hazards regression analysis with imputation by multiple chained equations of predictive factors for genitourinary toxicity-related admission following primary prostate EBRT

Characteristic	*N*	Univariable regression			Multivariable regression		
		HR	95% CI	*p* value	HR	95% CI	*p* value
Age, years	3243	1.01	1.00, 1.03	**0.010**	1.01	1.00, 1.02	0.2
Charlson Score	3243	1.06	0.99, 1.12	0.091			
Diabetes	3243	1.54	1.29, 1.82	**< 0.001**	1.28	1.08, 1.53	**0.004**
Obesity	3243	1.56	0.99, 2.46	0.076			
Hypertension	3243	4.74	1.18, 19.0	0.080			
Tumour stage	3243	1.00	0.79, 1.26	> 0.9			
Gleason score	3243	1.03	0.96, 1.10	0.5			
PSA	3243	1.00	1.00, 1.00	0.2			
Smoking	3243	2.73	2.30, 3.23	**< 0.001**	1.67	1.40, 2.00	**< 0.001**
Anticoagulation	3243	1.96	1.60, 2.40	**< 0.001**			
Urinary incontinence	3243	7.82	6.60, 9.27	**< 0.001**	3.95	3.28, 4.75	**< 0.001**
BOO and TURP.c	3243	3.58	3.02, 4.25	**< 0.001**	3.67	3.01, 4.46	**< 0.001**
BOO no TURP	3243	6.07	5.10, 7.23	**< 0.001**	5.87	4.80, 7.17	**< 0.001**
No BOO no TURP	3243	0.15	0.13, 0.17	**< 0.001**			
EBRT before 2009	3243	1.24	1.04, 1.47	**0.016**	0.87	0.72, 1.04	0.12
Dose, Gy	3243	1.00	0.99, 1.02	0.4			

Any statistically significant *p*-values were bolded

HR, Hazard ratio; CI, confidence interval; PSA, Prostate specific antigen; BOO, Bladder Outlet Obstruction; TURP, Transurethral Resection of Prostate; EBRT, External beam radiotherapy; Gy, Gray

## Discussion

This is one of few studies to evaluate the cumulative incidence of treatment-related genitourinary complications following radiotherapy for prostate cancer at a population level and the first in Australia. The high 10-year cumulative incidence (28.4%) of hospital admission due to treatment-related genitourinary toxicity exceeds previous estimates following primary EBRT [4, 6, 7]. The date of radiotherapy made a minimal difference in the 10-year cumulative incidence of genitourinary toxicity-related admission amongst patients in this cohort, and was not an independent predictor of genitourinary toxicity after adjustment for age, comorbidity, smoking and BOO in multivariable analysis (HR 0.87 [95% CI 0.72, 1.04], *p* = 0.12; Table 4). This is also the first Australian study to determine the volume of admissions and urological procedures for the management of radiotherapy treatment-related genitourinary complications at a population level. Greater than one-third of genitourinary toxicity-related hospital admissions occurred in the emergency setting. There were a significant number of admissions with a prolonged length of stay of ≥ 3 days. Whilst haematuria was the most common presentation, we are unable to confirm the diagnosis of radiation cystitis due to the limitations associated with administrative coding, we can infer the diagnosis of severe hemorrhagic radiation-induced cystitis occurred in 12/3351 (0.4%) of patients, with 8/3351 (0.2%) and 4/3351 (0.1%) patients requiring HBOT and surgical urinary diversion, respectively. A significant number of patients (18%) required an invasive urological procedure. There were significantly fewer hospital admissions and procedures amongst patients treated with EBRT after 2009, which may reflect improvements in radiotherapy techniques or the shorter follow-up in this group, which likely underestimated late toxicity.

Three large population-based studies have been published in this area with patients from the USA [17], Canada [6], and England [7]. A total of 307,252 patients were described [4, 6, 7]. However, like several other studies [18, 19], these studies did not include patient baseline oncological characteristics [6], or important treatment-related factors, including the dose and fractionation use in the radiation treatment used [6, 7, 17]. The study by Sheets et al., was the first study to demonstrate an increased risk of patients developing genitourinary toxicity following IMRT as compared to conformal radiation therapy, (absolute risk, 5.9 vs 503 per 100 person-years; relative risk, 1.12; 95% CI 1.03–1.20) [17]. Only one of these studies reported 5-year cumulative incidence of treatment-related genitourinary toxicity, which was determined to be 10.7 (95% CI 10.1–11.3) [7]. The estimate determined by the latter study was limited by missing values for the prostate cancer risk group (*n* = 5753) and radiotherapy treatment region (*n* = 3793) [7]. The other study reported a 22.2% (95% CI 21.7–22.7) 5-year cumulative incidence of admission for either genitourinary or gastrointestinal treatment-related complication and a 32.0% (95% CI 31.4–32.5) 5-year cumulative incidence of needing a urological procedure [6]. All three studies lacked a 60-month endpoint and this may have led to an underestimation of the late genitourinary toxicity events, as is the case with many other studies [19, 20]. The majority of studies of > 5-year genitourinary toxicity are not population-based, tend to focus on a narrower range of toxicity and have a shorter follow-up duration [9, 21].

Patients with bladder outlet obstruction without TURP before EBRT were at the highest risk of developing genitourinary toxicity after adjustment for age, diabetes, smoking, urinary incontinence and EBRT before 2009 (HR 5.87 [95% CI 4.8–7.17], *p* < 0.001; Table 4). Similarly, many other studies have also shown that pre-existing urinary symptoms can influence radiotherapy-related genitourinary toxicity [22–24]. TURP before radiotherapy demonstrated a protective effect against genitourinary toxicity amongst patients with bladder outlet obstruction in our study (HR 3.6, 95% CI 3.01–4.46, *p* < 0.001); however, other studies have shown TURP might deteriorate late urinary symptoms [25, 26]. Similarly, several other studies [23, 24, 27] have supported our finding that diabetes is an independent predictor of genitourinary toxicity in patients with prostate cancer treated with radiotherapy (HR 1.25, 95% CI 1.08–1.53, *p* < 0.004). Furthermore, the role of diabetes may be increasingly important in the era of dose-escalated (≥ 74 Gy) IMRT, as shown by Kalakota et al., who reported diabetes to be an independent predictor of late grade 3 genitourinary toxicity (RR 2.74, *p* = 0.004) in their multivariate analysis [28]. However, a few studies did not support the impact of diabetes on treatment-related genitourinary toxicity [29–31].

Less known is the impact of age on radiation-induced genitourinary toxicity, which may reflect physiological changes and altered clinical decision-making. Whilst we found that increased age was associated with significant lower cumulative 5-, 10- and 15-year EFS rates (*p* = 0.041, Table 4) in univariate analysis (HR 1.02 95% CI 1.01–1.03, *p* < 0.001), this did not retain significance in multivariable regression (*p* = 0.6). However, other studies have shown increased age to be an independent predictor of treatment-related genitourinary toxicity [6, 23, 32], including the study by Nam et al., which reported a higher incidence of hospital admission due to genitourinary toxicity (HR 1.007, 95% CI 1.003–1.010, *p* < 0.0001) amongst patients with prostate cancer treated with radiotherapy (*n* = 16,595) in a multivariable analysis performed in Cox proportional hazard modelling, adjusted for age and comorbidity treatment [6].

Similarly, whilst we found an increased risk of genitourinary toxicity amongst patients with a history of anticoagulation medication use on univariable analysis (HR 2.03 95% CI 1.67–2.49, *p* < 0.001), the significance was not retained in multivariable analysis (*p* = 0.3). However, in multivariable analysis, other studies have shown an increased risk of haematuria associated with anticoagulant use (RR 2.89, *p* = 0.01) [23].

Whilst we found that Charlson comorbidity score was not associated with genitourinary toxicity, in univariate analysis (HR 1.06, 95% CI 0.99–1.12, *p* < 0.091), the study by Nam et al. found that increased comorbidity, as measured by the Johns Hopkins University ACD Case-Mix System, was associated with a higher incidence of hospital admission in multivariate analysis (HR 1·08, 95% CI 1.07–1.09, *p* < 0.0001) [6].

Similarly, whilst we found no statistically increased risk of toxicity for patients with a history of hypertension (HR 3.91, 95% CI 0.98–15.7, *p* = 0.12) on univariable analysis, other studies have shown a positive association [30, 33]. Contrastingly, other studies have reported a protective effect of hypertension, suggested to be associated with antihypertensive medication intake [34], with Barnett et al. reporting a correlation with decreased risk of a poor urinary stream (HR 0.25, 95% CI 0.09–0.71, *p* = 0.009) [30].

Similarly, the data on dose-related genitourinary dysfunction has been controversial, and whilst some studies suggested a correlation between dose to the bladder and genitourinary toxicity [23, 24, 35–41], this has generally been unconfirmed by other authors [26], including the current study in univariable regression analysis (*p* = 0.4). This inconsistency may be due to confounding differences in treatment scheme (target volume, position during treatment, bladder volume variation, technique, dose), patient characteristics, grading scale and the length of follow-up [42–44].

Similarly, patients who received radiotherapy before 2009 had a higher 10-year cumulative incidence of admission for genitourinary toxicity (29% [26%, 31%] vs 19% [16%, 21%] *p* < 0.001; Table 3, Fig. 3). In addition, patients with EBRT before 2009 had more hospital readmissions for genitourinary toxicity (1879 [74%] vs 1354 [77%], *p* < 0.001), urinary retention (757 [43%] vs 287 [38%], *p* = 0.032) as well as more non-operative (*p* < 0.001) and minor-operative procedures (*p* < 0.001) compared with patients who received radiotherapy ≥ 2009 (Table 1). However, date of treatment before 2009 was not an independent predictor of hospitalisation for genitourinary toxicity, after adjustment for age, comorbidity, smoking and BOO (HR 0.87 [95% CI 0.72, 1.04], *p* = 0.12; Table 4).

Our study has several limitations. First, whilst the use of administrative data coding based on diagnostic and admission codes has been validated in other claims-based studies assessing severe pelvic adverse effects after radiotherapy [45], the number of genitourinary complications has likely been under-reported given the retrospective data-linkage methods used. For example, we would not have captured complications that are non-life-threatening (e.g., lower urinary tract symptoms from urethral stricture or bladder neck contracture) or which do not require further procedures. Furthermore, the sampling methodology used does not account for patients who may have had complications in other states. However, the study benefits from population-level data and longer duration of follow-up. In addition, we are unable to establish a causal link between radiation treatment and the reason for admission. These potential confounding factors may lead to the incorrect attribution of radiation-related toxicity in our data set, especially for late complications given the distant temporal relationship [19, 46]. The work presented here is descriptive and may motivate further investigations focusing on causal pathways, mechanisms of action and preventive strategies. Toxicity grades were unable to be reported, as these were not coded in administrative data. The study does not include radiation-associated secondary malignancy, gastrointestinal or other pelvic treatment-related complications (e.g., rectal and pubic symphysis fistula).

## Conclusions

Genitourinary complications after radiotherapy for prostate cancer are common. Although there continue to be significant advancements in radiotherapy techniques, patients and physicians should be aware of the risk of late toxicity when considering treatment options for prostate cancer. Further research is needed to identify predictive factors and develop models predicting late treatment-related genitourinary toxicity to improve pre-treatment counselling and enhance patient-centered decision making.

## Supplementary Information

Below is the link to the electronic supplementary material.

Supplementary file1 (DOCX 36 KB)

## Abbreviations

BOO: Bladder Outlet Obstruction
CTCAE: Genitourinary toxicity is defined by the CTCAE as the presence of any of the following adverse events, as explained in greater detail on the NCI Web site: bladder spasms, cystitis, genitourinary fistula, urinary incontinence, genitourinary leak, genitourinary obstruction, genitourinary perforation, prolapse of stoma, renal failure, stricture/stenosis, urinary electrolyte wasting, urinary frequency/urgency, urinary retention
GU: Genitourinary
Gy: Gray
PSA: Prostate-specific antigen
RTOG: Radiation Therapy Oncology Group. Grade > / = 3 toxicity is often used a proxy for serious toxicity that would necessitate hospitalisation for management. Published rates from large RCTS for *G* = / > 3 range from 6 to 13% [38, 47, 48]
TURBT: Transurethral Resection of Bladder Tumour
TURP: Transurethral Resection of Prostate
NCCN: National Comprehensive Cancer Network
