# Supplementary material for: Incidence of genitourinary complications following radiation therapy for localised prostate cancer
Source: World J Urol. 2022 Aug 11;40(10):2411–22. doi: 10.1007/s00345-022-04124-x (PMC9512751; doi:10.1007/s00345-022-04124-x)
Supplement: Supplementary file 1 — Supplementary file1 (DOCX 36 KB) [file 345_2022_4124_MOESM1_ESM.docx]

# Appendix 1

## Table 1. Treatment-related genitourinary complication-related hospital admission ACHI code

| **DESCRIPTION** | **ACHI Code** | **Category 1** |
| --- | --- | --- |
| Haematuria | R31 | Haematuria (10) |
| Recurrent and persistent haematuria | N02 | Haematuria (10) |
| Recurrent & persistent haematuria other | N028 | Haematuria (10) |
| Recurrent & persistent haematuria unsp | N029 | Haematuria (10) |
| Irradiation cystitis | N304 | Haematuria (10) |
| Congestion and haemorrhage of prostate | N421 | Haematuria (10) |
| Urethral stricture | N35 | Urinary obstruction(10) |
| Other urethral stricture | N358 | Urinary obstruction(10) |
| Urethral stricture unspecified | N359 | Urinary obstruction(10) |
| Other disorders of urethra | N36 | Urinary obstruction(10) |
| Urethral disorder unspecified | N369 | Urinary obstruction(10) |
| Oth atresia stenos urethra bladder neck | Q643 | Urinary obstruction(10) |
| Oth atrs stenos urethra & bladder neck | Q6439 | Urinary obstruction(10) |
| Postprocedural urethral stricture | N991 | Urinary obstruction(10) |
| Injury of urethra | S373 | Urinary obstruction(10) |
| Bladder-neck obstruction | N320 | Urinary obstruction(10) |
| Retention of urine | R33 | Urinary obstruction(10) |
| Mech comp urinary (indwelling) catheter | T830 | Urinary obstruction(10) |
| Stress incontinence | N393 | Urinary incontinence |
| Other specified urinary incontinence | N394 | Urinary incontinence |
| Unspecified urinary incontinence | R32 | Urinary incontinence |

## Table 2. Procedure categories

### 3A. Non-operative procedures

| **DESCRIPTION** | **ICD-10** |
| --- | --- |
| Bladder catheterisation (8, 10) | 3680000 |
| Bladder irrigation (8) | 9210100 |
| Irrigation of catheter | 9219500* |
| Passage of urethral sounds | 3730000* |
| Dilation of urethral stricture(10) (8) | 3730300 |

### 3B. Minor-operative procedures

| **DESCRIPTION** | **ICD-10** |
| --- | --- |
| Urethroscopy | 3731500 |
| Cystoscopy (10) | 3681200 |
| Cystoscopic urinary catheter exchange | 3680001 |
| Cystoscopic lavage of blood clots from bladder(10) | 3684200 |
| Endosc dest bladder lsn / tiss <= 2 cm | 3684003 |
| Endosc resec lsn / tiss bladder <= 2 cm | 3684002 |
| Inj/o para-urethral bulk, male incont | 3733901 |
| Internal urethrotomy(10) (8) | 3732401 |
| External urethrostomy | 3732402* |
| Optical urethrotomy(10) (8) | 3732700 |
| Balloon dilation urethral stricture | 9037100*, 9039400 |
| Endoscopic incision of bladder neck(8) | 3685400 |
| Endoscopic resection of bladder neck(8) | 3685402* |
| Percutaneous cystotomy [cystotomy] (8) (10) | 370110 |
| Ureteric dilation | 3680301, |
| Antegrade percutaneous procedure (nephrostomy, antegrade stent procedure) | 3662401, 3660800, 3660400, 3665000 |

### 3C. Major-operative procedures

| **DESCRIPTION** | **ICD-10** |
| --- | --- |
| Ureteric reimplantation(10) | 3658801 |
| Urethroplasty - single stage procedure | 3734200 |
| Urethroplasty, staged proc, first stg | 3734500 |
| Urethroplasty, staged proc, second stg | 3734800 |
| Insertion artificial urinary sphincter (8) | 3738700 |
| Revision of artificial urinary sphincter (8) | 3739000 |
| Replacement artificial urin sphincter (8) | 3739001 |
| Removal of artificial urinary sphincter (8) | 3739002 |
| Retropubic proc male stress incontinence(8) | 3704400 |
| Rev retropubic proc, male stress incont (8) | 3704403 |
| Div ureth slg foll stres incont proc (8) | 3734000 |
| Cystotomy [cystotomy] (10) | 3700801 |
| Laparoscopic cystotomy [cystotomy] (10) | 3700800 |
| List of codes for Urinary Diversion (Ileal conduit, cystectomy) (10) | 3660002, 3701400 |
| Formation of continent valve(10) | 3701100 |
